# Genetics of healthy aging and longevity

**DOI:** 10.1007/s00439-013-1342-z

**Published:** 2013-08-08

**Authors:** Angela R. Brooks-Wilson

**Affiliations:** 1 Canada’s Michael Smith Genome Sciences Centre, BC Cancer Agency, 675 West 10th Avenue, Vancouver, BC V5Z 1L3 Canada; 2 Department of Biomedical Physiology and Kinesiology, Simon Fraser University, 8888 University Drive, Burnaby, BC V5A 1S6 Canada

## Abstract

Longevity and healthy aging are among the most complex phenotypes studied to date. The heritability of age at death in adulthood is approximately 25 %. Studies of exceptionally long-lived individuals show that heritability is greatest at the oldest ages. Linkage studies of exceptionally long-lived families now support a longevity locus on chromosome 3; other putative longevity loci differ between studies. Candidate gene studies have identified variants at *APOE* and *FOXO3A* associated with longevity; other genes show inconsistent results. Genome-wide association scans (GWAS) of centenarians vs. younger controls reveal only *APOE* as achieving genome-wide significance (GWS); however, analyses of combinations of SNPs or genes represented among associations that do not reach GWS have identified pathways and signatures that converge upon genes and biological processes related to aging. The impact of these SNPs, which may exert joint effects, may be obscured by gene-environment interactions or inter-ethnic differences. GWAS and whole genome sequencing data both show that the risk alleles defined by GWAS of common complex diseases are, perhaps surprisingly, found in long-lived individuals, who may tolerate them by means of protective genetic factors. Such protective factors may ‘buffer’ the effects of specific risk alleles. Rare alleles are also likely to contribute to healthy aging and longevity. Epigenetics is quickly emerging as a critical aspect of aging and longevity. Centenarians delay age-related methylation changes, and they can pass this methylation preservation ability on to their offspring. Non-genetic factors, particularly lifestyle, clearly affect the development of age-related diseases and affect health and lifespan in the general population. To fully understand the desirable phenotypes of healthy aging and longevity, it will be necessary to examine whole genome data from large numbers of healthy long-lived individuals to look simultaneously at both common and rare alleles, with impeccable control for population stratification and consideration of non-genetic factors such as environment.

## Background

The basis of human longevity and healthy aging, and how to achieve these desirable phenotypes, remain among the principal challenges of biology and medicine. While an understanding of lifestyle and environmental factors will maximize our ability to prevent disease and maximize health in the general population, studying the genetic basis of longevity and healthy aging in exceptional individuals is providing important biological insights. In model organisms it has been possible to demonstrate effects of mutations in genes that can extend lifespan nearly tenfold (Ayyadevara et al. 2008). Studies of inbred lab strains and of natural genetic variants in model organisms including yeast and worms (Tissenbaum and Guarente 2002), flies (Paaby and Schmidt 2009) and mice (Yuan et al. 2011) have clearly implicated many specific genes in the lifespan of these organisms. Our understanding of human lifespan stands in contrast to this, with only one consistently replicable genetic association, APOE, observed to date in several genome-wide association scans (GWAS) of longevity-related traits. This may be because healthy aging and longevity are particularly complex traits, involving not only maintenance of long-term function but also absence or reduction of disease and other morbidities. It has been proposed that human lifespan is influenced not only by longevity assurance mechanisms and disease susceptibility loci but also by the environment, gene–environment interactions, and chance (Cournil and Kirkwood 2001). It will be important to understand the effects of environment (lifestyle) and of genetics, as well as how they interact to affect health and lifespan.

The importance of age and aging is underscored by the recognition that all common complex diseases increase with age. Questions remain about whether aging is the cause or effect of such diseases (Hekimi 2006). The study of desirable phenotypes like longevity and healthy aging has been referred to as ‘positive biology’ (Farrelly 2012). Its premise is that understanding the basis for such desirable traits may allow us to design interventions to improve human health.

This review was intended to summarize our current understanding of genetic factors affecting the phenotypes of longevity and healthy aging in humans, including the definition and heritability of these traits, and linkage, association, and sequencing studies. The surprising and novel findings that centenarians do not appear to have a relative lack of common complex disease risk alleles, and that some genetic variants appear to ‘buffer’ or protect against specific risk alleles, are discussed in detail. Shorter summaries of the findings related to somatic mosaicism and the promising study of epigenetics of aging are included for completeness.

### Aging, healthy aging, and longevity

The phenotypes used in studies of the genetics of human aging are usually lifespan (age at death), longevity (long life, usually defined as being a specific advanced age or older at the time of study), exceptional longevity (defined as attaining or exceeding a specific exceptional age), or healthy aging (a combination of old age and health, often defined as freedom from specific disorders or desirable performance levels on functional tests). Longevity studies focus on long-lived individuals (LLI), often centenarians aged 100 or more years. One advantage of such studies is the simplicity of phenotype definition. Healthy aging can be defined in various ways, usually with regard to reaching an at least moderately old age in the absence of certain diseases or disabilities, and/or in the presence of desirable traits such as intact cognition or mobility. Both types of studies should be differentiated from the study of the fundamental biological processes of aging (for example, cellular senescence).

A major difference between longevity and healthy aging studies is that the former focuses on lifespan, whereas the latter is focused on healthspan. Lifespan and healthspan are intimately related, however, and individuals who live exceptionally long also tend to be healthy for much of their lives. A landmark study of the health of supercentenarians (aged 110–119), semisupercentenarians (aged 105–109), centenarians (in this context aged 100–104), nonagenarians, and younger controls found that the older the age group, the greater the delay in onset of major disease (Andersen et al. 2012). Remarkably, for every category of increasing age, the hazard ratio for each of six disorders (cancer, cardiovascular disease (CVD), dementia, hypertension, osteoporosis, and stroke) was <1.0 relative to the next oldest group. This delay in disease development and postponement of cognitive and physical decline in the oldest group amounted to a compression of morbidity (Fries 1980). Based on these findings, Andersen et al. (2012) suggest that a realistic and practical limit of human lifespan is 110–115 years, close to that of the oldest documented person in the world to date, who lived to 122 (Robine and Allard 1998).

Women have a lower mortality rate than men at every age, and women live longer than men in most human populations. Any given exceptional age, therefore, is more exceptional for men than for women. As noted by Sebastiani and Perls (2012) 1 % of US women (but only 0.1 % of men) born circa the turn of the last century lived to be 100. Potential explanations for this difference include hormonal and immune differences, hemizygosity of the X-chromosome in men (which may allow manifestation of unfavorable sex-linked variants), and unrecognized confounders [reviewed in Newman and Murabito (2013)].

### The heritability of lifespan and related traits

Age at death in adulthood has a heritability of approximately 25 % (summarized in Murabito et al. 2012). A population-based study of 2,872 Danish twin pairs born between 1870 and 1900 found that the heritability of adult lifespan was 0.26 in men and 0.23 in women (Herskind et al. 1996). This cohort was not only population-based but nearly non-censored and, with follow-up for 94 years, encompassed essentially the entire human lifespan. Importantly, the heritability of longevity increases with greater age. The heritability of living to at least 100 has been estimated at 0.33 in women and 0.48 in men (Sebastiani and Perls 2012). Male and female siblings of US centenarians were 17-fold and eightfold more likely (compared with US Social Security data) to reach the age of 100, respectively (Perls et al. 2002). The increase in heritability of longevity at greater age is consistent between several studies. In over 20,000 Scandanavian twins, heritability of longevity was negligible from age 6–60, but increased with age thereafter (Hjelmborg et al. 2006). Long life was heritable in Icelanders aged over 70 years (Gudmundsson et al. 2000). The siblings of Okinawan centenarians show increased adult survival probability that starts at age 55 and increases with age (Willcox et al. 2006); the authors speculate based in part on absence of many age-related diseases from Okinawan (Bernstein et al. 2004) and other centenarians (Evert et al. 2003), that these individuals have genetic factors that confer resistance to such diseases and increase the likelihood of reaching exceptional old age. The estimation of heritability also depends on how it is studied; Murabito et al. (2012) note that the Framingham heart study cohorts give much greater estimates of heritability when longevity is studied as a dichotomous trait (36 % heritability for survival to 65 and 40 % for survival to 85), compared with 16 % heritability when age at death is treated as a continuous trait.

Clustering of longevity and healthy aging is observed in families. Parents of centenarians born in approximately 1870 were sevenfold more likely than their contemporaries to have lived to age 90–99; offspring of centenarian parents showed lower prevalence of age-related disease than age-matched control groups (Atzmon et al. 2004). Exceptional familial clusters of extreme longevity have also been reported (Perls et al. 2000). Healthy aging is also heritable. Reed and Dick (2003) defined ‘wellness’ in male twins as achieving the age of 70 free of heart attack, coronary surgery, stroke, diabetes or prostate cancer, and showed that this trait had a heritability exceeding 50 %.

Environment and lifestyle likely constitute much of the remaining influence on human lifespan and healthspan. These factors have varied greatly over time and may not reflect the extrinsic factors that will affect the lifespan of babies born today. Many members of the elderly and centenarian cohorts under study today lived through times of caloric restriction (e.g., the Great Depression) and grew up before the use of antibiotics and vaccines became commonplace. The selective pressures that influenced their mortality are not identical to those experienced by later generations, and this is an important consideration for study design.

### The importance of study design

Phenotype definition is particularly important in genetic studies; it affects the interpretation and meaning of results, and the ability to compare to the results of other studies. Studies of longevity can include extreme longevity (defined as living beyond a specific extreme age) or age at death. Studies of healthy aging may use age to disease onset, successful aging or wellness (which can also have a variety of definitions), or other phenotypes (Manolio 2007). Linkage or family-based association study designs, longitudinal cohort studies, or case/control designs have been used. Family-based designs have the advantage of being robust to population stratification. Longitudinal cohorts have the advantage of limiting sampling bias, but take time and due to practical limits of size may not contain many individuals of extreme age. Sample size is a consideration for all these study designs. To date, the largest studies of LLI are in the low thousands of subjects; this is much smaller than the largest studies of common complex diseases (which now include over 100,000 subjects), despite the likely similar modest size of many of the genetic factors being sought.

Choice of a comparison group to contrast with exceptionally long-lived or exceptionally healthy elderly individuals is also critical. Health data for LLI can be compared with archived data for deceased individuals of the same birth cohort, but DNA samples from an ideal comparison group (such as their birth cohort) are not available. Case/control molecular genetic studies of long-lived or healthy aged individuals often compare elderly cases to younger controls. Potential pitfalls of such studies include inadequate detection and control for population stratification, particularly for populations that have experienced immigration of different ethnicities over time (Nebel and Schreiber 2005). The use of principal components analysis (Price et al. 2006) or genomic controls (Devlin and Roeder 1999) can mitigate this problem, as can the conduct of studies within specific ethnic groups (Barzilai et al. 2001). In the case/control design, the control group is also expected to contain individuals who will go on to become equivalent to cases; their presence in the control group reduces power. Environmental factors must be acknowledged in such studies as potential confounders; inevitably, the cases and controls have lived in different times and experienced different lifestyles. A way to mitigate some of these problems; however, is to choose controls that are no older than 50 (Halaschek-Wiener et al. 2008) because in modern day developed countries, mortality before age 50 is minimal. Choosing a comparison group <50 years of age makes the control group essentially an ‘unselected’ group with regard to mortality from age-related diseases. Choosing a control group in their 70 or 80, however, would exacerbate this issue, as the control group would fail to include individuals who died in their 50’s or 60’s.

Several studies, such as the Longevity Gene Study (Barzilai et al. 2001), the Leiden Longevity Study (LLS) (Mooijaart et al. 2011), the New England Centenarian Study (NECS) (Terry et al. 2004), and the Long Life Family Study (LLFS) (Newman et al. 2011) include comparisons of the offspring of LLI (who are assumed to have inherited some longevity factors) to contemporary age-matched controls. They have observed that the offspring of LLI have more favorable blood lipid profiles (Barzilai et al. 2001; Newman et al. 2011) and lower prevalence of hypertension and metabolic and cardiovascular disease (Atzmon et al. 2004; Westendorp et al. 2009; Newman et al. 2011) and all-cause mortality (Terry et al. 2004) than age-matched controls. Comparison of the offspring of LLI with their contemporaries controls for cohort effects such as variation in BMI in human populations over time; it has the limitation, however, of under-estimating the difference in phenotypes and genotypes that would presumably be observed if the LLI could be compared with their largely long-deceased birth cohort.

### Linkage studies of longevity and healthy aging

Linkage studies of long-lived sibships or extended pedigrees with exceptionally long-lived individuals have identified several putative and one replicated longevity linkage. In 2001, the NECS (Puca et al. 2001) reported a 10-cM sib-pair based linkage scan of 308 individuals in 137 sibships with exceptional longevity (defined as having a proband of at least 98 and a 91-year-old male or 95-year-old female sib). They found significant evidence for linkage of longevity to a region around D4S1564. Suggestive support for this region was obtained through analysis of 95 concordant pairs of fraternal male twins with a wellness phenotype (age at least 70 with no overt CVD or prostate cancer) (Reed et al. 2004). Initial convergence of two linkage studies with very different phenotypes led to excitement about this region and its suspected role in longevity and health. Subsequent study of the region focused in part on a regional biological candidate gene, microsomal triglyceride transfer protein (MTP), identified through haplotype analysis (Geesaman et al. 2003).

In 2010 a larger and higher density linkage study (Boyden and Kunkel 2010) expanded on the initial NECS resource, with a genome-wide linkage study of 279 families with multiple long-lived sibs 90 years and older, including 129/137 of those previously described (Puca et al. 2001). A limitation of this study was the use of expected life span (estimated from age- and gender-specific life expectancies) for the 70 % of subjects who were still living. This analysis of 9,751 SNPs found just-significant LOD scores at 3p22-24 and 9q31-34, as well as modest evidence for linkage at the original site, 4q22-25 and possibly at 12q. A larger study (Kerber et al. 2012) replicated the linkage of 3p22-24 to extreme longevity and identified possible additional loci. Working with 732 subjects from the Utah population database and database and population controls, including 433 Caucasian individuals aged 86–109 who showed a phenotype including both excess individual longevity (the difference between observed and expected lifespan) and excess familial longevity (a weighted average of excess longevity for all family members), they used a linkage screen with 1,100 microsatellite markers to identify a strongly suggestive peak at 3p at the same position as Boyden and Kunkel. Meta-analysis of linkage in the Utah and New England data sets supported linkage at the chromosome 3 locus. Other linkage peaks were observed in the Utah data at 18q23-24, 8q23, and 17q21; meta-analysis provided additional support but not outright replication for 8q, 9q, and 17q. The new data; however, did not support linkage to chromosome 4 or chromosome 12. Larger sample sets and denser and more informative linkage analyses were pointing away from the original chromosome 4 linkage observation, and converging instead most strongly at 3q24-22.

Two linkage studies of successful aging in Amish individuals over 80 years of age within a single 13-generation pedigree showed linkage to chromosomes 6, 7, and 14, different regions than those found in the longevity linkage studies. Successful aging was defined as cognitively intact and without depression, high functioning, and satisfied with life. These studies (Edwards et al. 2011; Edwards 2012) analyzed 263 cognitively intact Amish over 80 years old (74 successfully aged and 189 normally aged) within 12 sub-pedigrees using 630,309 autosomal SNPs. Linkage was found at 6q25-27, as well as association of a SNP, rs205990, in the interval linked to the ‘successfully aged’ phenotype. The chromosome 6 linkage identified in the Amish is different from those identified in the Utah and New England studies; this may reflect the different phenotype, or may be due to genetic factors specific to the Amish founder population.

The largest linkage study to date was done in the multi-site European Genetics of Healthy Aging (GEHA) Study, which looked at 2118 European full sib-pairs over 90 years old (Beekman et al. 2013). GEHA found linkage at 4 regions: 14q11.2, 17q12-q22, 19p13.3-p13.11, and 19q13.11-q13.32. The chromosome 14 linkage is at a different site from that observed in the Amish study; the large chromosome 17 region overlaps the 17q21 locus observed by Kerber et al. Fine mapping of these linkage regions using GWAS data in a subset of 1228 unrelated nonagenarians and 1907 controls identified a SNP near *APOE* at the 19q locus as significantly associated with longevity. Apolipoprotein E (apoE) isoforms are known risk factors for cardiovascular disease (CVD) and Alzheimer disease (AD), likely due to their involvement in inflammation, elevated lipid levels, and oxidative stress (Huebbe et al. 2011). ApoE has three main isoforms: apoE2, apoE3 and apoE4. Combined modeling in the GEHA study showed that *APOE4* (*p* = 0.02) and *APOE2* (*p* = 1.0 × 10^−5^) account for the linkage at 19q. The *APOE* linkage was characterized by absence of *APOE4*, but enrichment for* APOE2* among the nonagenarians. In this study the *APOE2* allele is the stronger association, and the authors refer to* APOE* as a longevity gene.

The multiple linkage signals observed in these studies likely indicate genetic heterogeneity of longevity and healthy aging in human populations. Interestingly, the GEHA study observed heterogeneity among its multiple geographic regions; Northern European subjects contribute most to some of the linkage peaks they observe, including the* APOE* locus. Gender-specific effects were also observed, with a male-specific linkage peak at 8p and female-specific ones at 15q and the 19q* APOE* locus (Beekman et al. 2013). While the lack of association at the other linkage regions in the GEHA study may be due to power limitations, it could also imply that multiple rare or ‘private’ variants contribute to linkage but not association at these loci.

### Candidate gene association studies

Candidate genes examined for association with longevity or healthy aging or related phenotypes fall into several categories. They include genes nominated based on observations of lifespan extension in model organisms; and genes involved in lipid metabolism, immune response and inflammation, stress response, and others. Candidate genes tested for association with longevity and related phenotypes have been the subject of several excellent reviews (Christensen et al. 2006; Wheeler and Kim 2011; Ferrario et al. 2012; Newman and Murabito 2013); an exhaustive listing is beyond the scope of this review.

Of the candidate genes assessed for association with longevity, variants in *APOE* and *FOXO3A* have been most consistently replicated, though some candidate genes have been associated with longevity phenotypes in more than one population but not in all populations tested; many more have been associated in a single study but failed to replicate in others (reviewed by (Christensen et al. 2006)). In a study of 1,344 healthy Italians aged 22–90, *APOE4* was found at lower frequency and *APOE2* at higher frequency in elderly and centenarians than in younger individuals (e.g., Seripa et al. 2006); *APOE2* is a putative protective factor in this context and *APOE*4 can be considered a ‘frailty’ allele (Gerdes et al. 2000). *FOXO3A* is a homologue of the *C. elegans Daf*-*16* gene that is important in control of lifespan in the worm (Hsin and Kenyon 1999); it is part of the insulin/IGF1 signaling pathway. *FOXO3A* variants have been associated with longevity in many populations (reviewed in Wheeler and Kim 2011).

Additional genes show promise of great relevance to healthy aging. A variant at *CETP*, for example, though inconsistently associated with longevity in different populations (reviewed by (Christensen et al. 2006)), in 213 Ashkenazi Jewish individuals of average age 98 is associated not only with longevity but also with additional aging-related phenotypes including a desirable lipid profile (Barzilai et al. 2003) and preservation of cognitive function (Barzilai et al. 2006). Other recent studies with extensive replication data are also encouraging. Association of a SNP in a heat shock factor gene, *HSF2* with all-cause mortality was seen in the longitudinal Rotterdam Study (5,974 participants and 3,174 deaths), with replication in eight population-based cohorts (Broer 2012).

Other candidate genes have been associated with longevity or healthy aging phenotypes in some but not all studies. MTP, identified as a regional candidate at the 4q25 locus, failed to show replication of association with longevity in larger studies of approximately 1500 LLI each (Beekman et al. 2006; Bathum et al. 2005; Nebel et al. 2005). Progeria genes have shown association with longevity in some studies. A haplotype of SNPs at* LMNA*, the gene that is mutated in Hutchinson-Gilford progeria, was associated with long life (age >95 years) in 873 LLI and 443 controls, and remained significant upon meta-analysis of 3,619 subjects from four independent samples (Conneely et al. 2012). Polymorphisms at* WRN* have shown inconsistent associations with age (Castro et al. 2000; Kuningas et al. 2006). Sirtuins mediate the effects of caloric restriction, a non-genetic factor known to increase life span in many organisms. The effect of polymorphisms in sirtuin genes (*SIRT1-7*) on longevity and age-related diseases was reviewed by Polito et al. (2010). There is evidence that variants in* SIRT3* (Rose et al. 2003) are associated with longevity. A functional promoter variant at DNA repair gene *EXO1* was associated with longevity in female centenarians (Nebel et al. 2009), but tagSNPs in the gene showed no association with longevity in men (Morris 2013).

Given the multifactorial nature and likely genetic heterogeneity of healthy aging and longevity, as well as environmental influences on these complex traits, it may not be reasonable to expect that replication of candidate gene studies would be uniform between populations. Reasons for lack of replication include limitations of sample size, rarity (low minor allele frequency) of actual variants, and small true effect sizes. Poorly designed or under-powered studies will result in false positives that legitimately fail to replicate. For studies of longevity and healthy aging, in particular, differences in phenotype or type of study will also result in findings that are non-uniform between studies. While larger case/control studies are frequently suggested as a solution to the limitations of present-day association studies, combining data from populations with different lifestyles and genetic backgrounds, even if well-matched for ethnicity, may obscure true association signals.

### Genome-wide association studies

To date, SNPs in or near *APOE* are the only ones to achieve genome-wide significance (GWS, generally *p* ≤ 5 × 10^−8^) in genome-wide association studies (GWAS) of lifespan-related traits. In three GWAS of long-lived individuals vs. younger controls, *APOE* was significantly associated with longevity at the genome-wide level. The first of these included 763 long lived (94–110 years) and 1,085 control (45–77 years) from German biobanks and replication in an independent set of German samples (754 cases aged 95–108, 860 controls aged 60–75) (Nebel et al. 2011). Only rs4420638 near *APOC1* and in linkage disequilibrium (LD) with *APOE* achieved GWS. GWAS of 403 unrelated nonagenarians (average age 94) from longevity families in the LLS vs. 1,670 controls (average age 58) showed similar results (Deelen et al. 2011a). Only one of 62 SNPs carried forward to meta-analysis with 4,149 nonagenarian cases and 7,582 younger controls from the Rotterdam study, the Leiden 85+ study and the Danish 1905 Cohort reached GWS, rs2075650 at *TOMM40* near *APOE*. Meta-analysis of the *APOE2* and *APOE4* SNPs showed significant associations of both SNPs with longevity, with E2 being protective of long life (OR 1.31, CI 1.17–1.46, *p* = 1.35 × 10^−6^), and E4 being deleterious (OR 0.62, CI 0.56–0.68, *p* = 1.33 × 10^−23^). A third longevity GWAS (Sebastiani et al. 2012) included three phases: a discovery phase with 801 New England centenarians (aged 95–119, many with a family history of extreme longevity) vs. 914 controls genetically matched by means of principal components analysis; a first replication in 253 centenarians (89–114) vs. 341 genetically matched controls; and a second replication with 60 additional centenarians (100–114) and unmatched controls. Of 243,980 SNPs analyzed only one, *TOMM40* SNP rs2075650 near *APOE*, reached GWS. Inverse association of* APOE4* with longevity (*p* = 5.3 × 10^−3^) was also detectable in the Southern Italian centenarians study (SICS) of 440 LLIs aged 90–109 and 553 young controls aged 18–45 (Malovini et al. 2011), despite the known lower frequency of the E4 allele in Southern, as compared with Northern, Europe (Haddy 2002).

Other GWAS of lifespan-related phenotypes revealed no associations that were significant at the genome-wide level. A GWAS of the Framingham health study (Lunetta et al. 2007) (258 Original Cohort and 1,087 Offspring individuals, members of the 330 largest families in the study) revealed no GWS SNPs for any of five aging-related phenotypes. Newman et al. (Newman et al. 2010) meta-analyzed four cohort studies in the cohorts for heart and aging research in genomic epidemiology (CHARGE) Consortium for survival to at least 90 years of age. Cases were 1,836 people who achieved survival to at least 90; controls were 1,955 participants who died aged 55–80. SNPs were genotyped and imputed in subjects of European ancestry, with systematic elimination of outliers and correction for population stratification. Replication was carried out in the LLS (950 long-lived probands and 744 partners of their offspring and 680 blood bank donors) and the Danish 1905 Cohort Survey (2,262 long-lived participants and 2007 Danish twin study controls aged 46–68). No SNPs reached genome-wide significance.

Walter et al. (2011) conducted a meta-analysis of GWAS of nine longitudinal cohort studies in the CHARGE Consortium, including 25,000 unselected people of European ancestry. They analyzed two continuous traits, all-cause mortality, and event-free survival (where ‘event’ was defined as myocardial infarction, heart failure, stroke, dementia, hip fracture, or cancer). No SNPs reached GWS for either phenotype. SNPs near *APOE* reached only nominal significance in the CHARGE study (Walter et al. 2011), in contrast to the results of GWAS of centenarians, in which *APOE* has been a significant and replicable finding. The CHARGE meta-analysis contained few extremely old individuals, and so in comparison with centenarian studies or those targeting long-lived healthy individuals, has examined earlier mortality and events, a different phenotype. The Framingham Study GWAS (Lunetta et al. 2007), which also showed no GWS SNPs also represents a much younger group, on average, than studies of oldest old or centenarians. This may mean that different genes and variants may come into play in different phases of aging, with *APOE* being most relevant at older ages. Earlier mortality is often related to lifestyle as well, and the heritability of aging is lower at younger ages, as described above.

A genome-wide association study of copy number variants (CNVs) in the Rotterdam study RS1 cohort, with replication in the RS2 cohort and the FHS, found that large common deletions are associated with mortality (vs. survival) at old age (Kuningas et al. 2011a). They tested 312 common CNV regions and measures of CNV burden for association with mortality during follow-up. A higher burden of CNVs of 500 kb or more in size was associated with mortality. Two specific regions were also associated with mortality, 11p15.5 and 14q21.3. The 11p15.5 association, which would survive Bonferroni correction for 312 tests, includes insertions and deletions which were analyzed together relative to non-carriers; it contains 41 genes including some related to longevity or complex diseases. The 14q21.3 region contains no genes and is characterized only by deletions. Runs of homozygosity, which can indicate presence of recessive loci, were not associated with survival to old age in this cohort (Kuningas et al. 2011b).

Analyses of phenotypes that may influence long-term good health have also been undertaken. Personality traits are associated with healthy aging and longevity (Terracciano et al. 2008). In the LLFS, a GWAS of five personality factors in 583 families with 4,595 individuals and replication in 1,279 other subjects identified a locus associated with agreeableness, and identified several significant age × SNP interactions that may affect longevity through effects on personality (Bae et al. 2013).

In contrast to the results of longevity GWAS, GWAS of common complex diseases have revealed hundreds of SNPs associated with cancers, CVD, diabetes and other age-related diseases, albeit with increasing numbers of associations found with increasing GWAS size. One explanation for this may be that the phenotypes of healthy aging and longevity may be much more complex than those of these complex diseases, in part because they often (depending on phenotype definition) involve absence of specific complex diseases. If GWAS studies of survival to elderly ages are even more confounded by environmental (E) factors than GWAS of diseases, combining studies from different populations in pooled or meta-analyses may complicate the E effects even more. In studies of older individuals, it is particularly hard to control for E factors experienced over many decades of life.

### Effects of variants that do not achieve *p* ≤ 5 × 10^−8^

SNPs at only one locus, *APOE*, have achieved Bonferroni-corrected levels of GWS in GWAS of longevity. By current standards these GWAS, which involved fewer than 1,000 centenarians, or a few 1,000 nonagenarians, are modest in size. Larger GWAS may in theory allow additional SNPs to achieve this threshold. There are other indications, however, that support the idea that SNPs that do not reach this threshold of GWS may be biologically important, either individually or through their joint effects. Several studies used a variety of techniques to analyze collections of nominally longevity-associated SNPs to determine if they act in concert to affect lifespan.

In the Framingham study GWAS of 5 aging-related phenotypes (Lunetta et al. 2007) observed that SNPs in some candidate genes, including SNPs near the Werner syndrome gene *WRN* and *FOXO1A*, as well as *GAPDH*, *KL*, *LEPR*, *PON1*, *PSEN1*, and *SOD2* were associated with age at death. Kulminski and Culminskaya (2011) used Framingham Affymetrix 50 K SNP data to perform GWAS of four endophenotypes (CVD, cancer, systolic blood pressure, and total cholesterol) to identify 63 SNPs that were associated at *p* < 10^−6^ with at least one endophenotype. 76 genes at or near these SNPs were enriched in terms of Gene Ontology annotations related to aging-relevant processes. Yashin et al. (2010) hypothesized that lifespan depends on the number of small-effect longevity alleles present in individual genomes. They re-analyzed Framingham 550 K SNP data and identified 169 SNPs associated at *p* < 10^−6^. The number of these SNPs carried by an individual correlated with lifespan and explained 21 % of its variance; in contrast, randomly chosen SNPs did not correlate with lifespan.

Gene set analysis of GWAS data from the LLS and Rotterdam studies was used to show that genes in the insulin/IGF-1 signaling (IIS) and telomere maintenance TM pathways are associated with longevity (Deelen 2011b). 1021 and 88 GWAS SNPs were identified within 10 kb of 68 IIS and 13 TM genes, respectively. Both pathways were associated with longevity. Nine IIS genes (*AKT1*, *AKT3*, *FOXO4*, *IGF2*, *INS*, *PIK3CA*, *SGK*, *SGK2,* and *YWHAG*) and one TM gene (*POT1*) were the main determinants of the association.

Sebastiani et al. (2012) constructed a model in which 281 SNPs showed 89 % sensitivity and 89 % specificity to predict longevity in their GWAS Discovery set, and 58–61 % specificity and 58–85 % sensitivity in independent sets. They call this a ‘genetic signature of exceptional longevity’. These SNPs explain nearly 20 % of the heritability of extreme longevity. They find that the *TOMM40* SNP near *APOE* alone has poor predictive value; removing it from the model reduces specificity and sensitivity by only 1 %. The 281 SNPs include 137 in 130 genes, including *LMNA*, *WRN*, *SOD2*, *CDKN2A*, *SORCS1* and *SORCS2*, and *GIP*. This set of 130 genes is highly and significantly enriched for those related to Alzheimer disease (38 genes), 42 related to dementia, 38 to tauopathies, 24 to CAD, and several to neoplasms.

GWAS of the SICS Study of 410 LLI and 553 younger controls identified 67 SNPs that reached a permutation-defined level of genome-wide significance of *p* < 10^−4^ (Malovini et al. 2011). Among them was rs10491334 at the calcium/calmodulin-dependent protein kinase IV (CAMKIV) that replicated in 116 additional LLI and 160 controls. Malovini et al. demonstrate that CAMK4 phosphorylates and activates survival proteins FOXO3A, AKT, and SIRT1. Homozygous carriers of the minor allele had lower CAMKIV protein expression and were under-represented among LLI’s, consistent with a deleterious effect of this allele on longevity.

The biological relevance of other SNPs besides those at *APOE* is also strongly supported by similarities between the results of human GWAS and mouse lifespan studies. Eight of the ten top CHARGE SNPs detected by GWAS, but which did not achieve GWS, correspond to mouse lifespan quantitative trait loci (QTL) (Murabito et al. 2012). These studies connect GWAS findings that do not reach GWS with many genes that are relevant to aging or age-related diseases. In several cases, this convergence with genes of biological interest is statistically unlikely to be due to chance and is likely to reflect the presence of true association signals that are not consistent enough to be replicated predictably as candidate genes or achieve GWS, or have effects that are too subtle to be detected individually. Such potential true signals may be more affected by ‘E’ factors than those that have been replicated, i.e., *APOE* and *FOXO3A*. As pointed out by Yashin et al., the same sets of variants would not be expected to work in all populations because of differences in environment (Yashin et al. 2010).

### The extremely long-lived do not lack risk alleles for common complex diseases

Several recent studies have shown that centenarians do not carry smaller numbers of risk alleles for common complex diseases than average people. In an important paper in 2010, Beekman et al. (2010) studied two case/control collections: (1) 723 nonagenarian siblings (mean age 94) from the LLS vs. 721 unrelated younger controls (mean age 52), and (2) 979 long-lived individuals over 85 (mean age 87) from the pop-based Leiden 85+ study vs. 1,167 younger controls (mean age 41) from the Netherlands Twin Register. They looked at 30 SNPs known to be associated with CVD, cancer or type 2 diabetes (T2D). The cases and controls each carried an average of 27 disease risk alleles. The distribution of risk alleles was the same in elderly and young subjects. Beekman et al. note that “GWAS-identified disease risk alleles do not compromise human longevity” and suggest that a lack of rare disease factors, or the presence of protective factors, is at work in the long-lived individuals. It is important to note, however, that CVD, cancer, and T2D are diseases that have very clear lifestyle components and that part of the effect could be due to lifestyle differences.

Mooijaart et al. (2011) extended this observation the following year, showing that “SNPs associated with T2D and identified by GWAS are not major determinants of the beneficial glucose tolerance that characterizes familial longevity.” They compared the offspring of the LLS long-lived individuals with the offspring’s spouses and other controls. The LLS offspring had a better metabolic profile and better glucose tolerance than same-age controls, although the frequency of 15 known T2D SNPs did not differ between the two groups. When individuals were compared within each group, however, glucose levels did correlate with the number of T2D SNPs. They speculate that the LLS offspring may have protective factors that improve their metabolic profile and glucose tolerance in spite of the presence of T2D GWAS SNPs. This comparison, using same-age groups of individuals, clearly points to protective genetic factors contributing to preservation of a healthy phenotype, rather than lifestyle and environmental factors that should be very similar (at least in adulthood) between the offspring and their spouses.

Sebastiani et al. (2012) also noted that there was not a substantial difference in the numbers of 1,214 known disease-associated SNPs in centenarians and controls. A similar observation was made in their whole genome sequence data from one male and one female supercentenarian (Sebastiani et al. 2011).

These important and perhaps surprising results show that extreme longevity, and the long-term good health that often accompanies it, is not incompatible with the presence of many disease risk alleles. At least for the common SNPs associated with common complex diseases, it is not the absence of ‘bad’ alleles, but more likely the presence of ‘good’ alleles that influences longevity, though effects of ‘good’ environmental factors may also contribute. Protective factors of some kind may allow these risk variants to not be manifest. These results also have implications beyond the study of longevity—in an age when substantial effort is being invested in personalized disease risk prediction, the presence of many disease alleles that are non-penetrant in some individuals potentially complicates predictions of disease.

### Do ‘good’ variants protect against ‘bad’ ones?

One mechanism for a lack of effect by an undesirable allele is the ‘buffering’ mechanism explored by Barzilai et al. They propose that some individuals who show exceptional longevity may do so despite the presence of unfavorable alleles because those alleles are buffered by favorable alleles in other genes (Bergman et al. 2007). They suggest that buffering gene variants (longevity variants) will show a monotonic increase in frequency from early old age (65) to later ages; examples of buffering genotypes are *CETP* VV, *APOC3* CC, and a +2019 deletion in *ADIPOQ*. Buffered alleles, in contrast, should show a U-shaped frequency curve, higher at younger ages, dipping low in early old age, and then increasing in the exceptionally old (who have the ‘buffering’ protective genotype that allows disease-related variants to accumulate); examples of buffered genotypes are heterozygotes for deleterious alleles of *KLOTHO* and *LPA*. Importantly, Bergman et al. use a cross-sectional study design, with 1,200 subjects in their 6–11th decades of life to show experimental support for the buffering hypothesis; their data support the idea that *CETP* VV genotype buffers the deleterious effects of an* LPA* genotype. They show a genetic interaction between *CETP* genotype and *LPA*; *LPA* heterozygotes with the *CETP* IV/II genotypes monotonically decrease in frequency with age, but those in *CETP* VV individuals increase from age 70 onward. They argue that case/control analyses are insufficient to reveal this effect because it does not reveal the shape of the allele frequency × age curve.

Earlier observations are also explained by a buffering mechanism. De Benedictis et al. (1998) described an age-related convex trajectory of a 3′*APOB*-VNTR genotype that they interpret as consistent with crossing mortality curves relevant to subgroups of individuals with different genotypes. A X-sectional study of 800 healthy aging subjects from 18 to 109 years free of clinically apparent disease genotyped variants in *APOA1*, *APOC3*, and *APOA4* (Garasto et al. 2003). They noted that an allele of *APOA1* that correlated with higher serum LDL-C was paradoxically increased in frequency in the oldest old. The authors called it “another genetic paradox of centenarians.” While this observation could reflect population stratification in the different age groups, it may also be due to the U-shaped curve of a buffered gene.

The buffering mechanism may also explain some of the inconsistency in the findings for *MTP*. Huffman et al. (2012) find that *MTP* CC is a deleterious genotype that is buffered by any of three longevity genotypes of *CETP*, *APOC3,* or *ADIPOQ*. *MTP* CC shows a U-shaped curve, declining ages 55–85, and then dramatically increasing in those who live 90 or more years. If this *MTP* genotype is observed at high frequency in centenarians, but only in the presence of specific protective variants, this may in part explain why the linkage at chromosome 4 was not observed consistently between studies.

Buffering has been described in model organisms. The heat-shock protein Hsp90 is known to buffer genetic variation in *Drosophila*, allowing it to accumulate under neutral conditions (Rutherford and Lindquist 1998). Such a gene is known as a phenotypic capacitor, and it masks the presence of phenotypic variation. It is interesting to speculate that protective genetic variants carried by centenarians may be capacitors for the disease risk variants we now know they carry at, on average, the same frequency as other people. Identification of buffering/capacitor genes and study of their function will be necessary to understand the longevity phenotype. It will also be important to determine if such capacitors operate in healthy aging as well as extreme longevity. Because such variants are likely rare, intensive study of rare individuals at the upper ends of the human lifespan and healthspan, perhaps by whole genome sequencing and examination of unusual variants they carry, is paramount.

The interaction between buffering and buffered genes and genotypes also has implications for study design. The exquisite studies carried out by Barzilai’s group are done in a single well-defined ethnicity, Ashkenazi Jewish individuals. Since a buffered gene will only show a distinctive U-shaped curve in the presence of its buffer, and a buffer may only be advantageous in the presence of a deleterious gene that it buffers, this underscores the importance of avoiding population stratification in such studies. If some of the associations detected to date in case/control studies of healthy aging and longevity are actually underlain by genotypes with U-shaped curves, the choice of ages for the cases and controls will greatly affect whether an association is detected, and may explain some failures of associations to replicate. Finally, the concept of buffering genes has implications for the use of centenarians, or exceptionally healthy elderly individuals as super-controls for disease studies; if the exceptional elderly are healthy because of a protective factor rather than lack of a disease allele, their use as an extreme comparison group may not necessarily be helpful.

### Do differences in lifestyle affect these studies?

Given that lifestyle is expected to have a greater impact than genetics on healthy aging, it seems unlikely that differences in lifestyle are *not* confounding association studies of longevity and healthy aging. It is challenging to quantify lifestyle in an optimal comparison group for, for example, centenarians. Younger control groups inevitably have different lifestyles than the elderly had at their age. For example, the CHARGE consortium (Newman et al. 2010), which compared individuals who survived to at least 90 to those who died aged 55–80, found that the younger controls had higher rates of smoking.

The Longevity Gene Study overcame the birth cohort limitation using pre-existing lifestyle data from 3,164 NHANES controls of the same birth cohort as 477 Ashkenazi Jewish individuals aged 96–109 (Rajpathak et al. 2011). They found no obvious differences in lifestyle and suggested that the long-lived individuals may interact with lifestyle factors differently than others. This study, however, did note subtle differences between the long-lived and comparison groups. They saw significantly fewer obese men, more overweight women, and fewer obese women in the long-lived group; in addition, more control men smoked. These differences, combined with recall limitations of the long-lived group, imply that this analysis may have missed many small lifestyle differences that could add up to substantial health differences. It will likely be difficult to take into account all but the largest lifestyle factors when planning GxE studies of longevity and healthy aging. Biomarkers of exposures may vary not only with exposure but also over time, complicating the use of such methods for these phenotypes.

### Association studies of mitochondrial variants

Mitochondria are thought to be important to aging due to their key roles in oxidative phosphorylation, cell metabolism, and apoptosis. A relationship of variation in the mitochondrial genome with health and/or longevity is implied by the observation that age at death correlates more closely with the age at death of a person’s mother more so than that of the father (Brand et al. 1992). Associations of mitochondrial genome sequence variants or haplogroups (combinations of specific variants that correlate with specific populations) with healthy aging or longevity have been noted in many populations including, for example, Italian (De Benedictis et al. 1999), Japanese (Tanaka et al. 1998), Amish (Courtenay et al. 2012), Chinese Uygur (Ren et al. 2008), Costa Rican (Castri et al. 2009), Ashkenazi Jewish (Iwata et al. 2007), Irish (Ross et al. 2001), and Finnish individuals (Niemi et al. 2003). The associations observed are inconsistent between populations and do not involve the same variant or haplogroup. This lack of consistency may be due in part to the relatively small size of many of these studies. Three common problems have been noted about such studies: inadequate matching of cases and controls, inadequate correction for multiple tests, and undetected population stratification (Shlush et al. 2008).

Interestingly, when the frequencies of different mitochondrial haplogroups are plotted for Italian individuals aged 20 to over 100, the curve shapes observed include monotonic increase for haplogroup J, and a U-shaped curve for haplotype H (de Benedictis et al. 2000), reminiscent of the ‘longevity’ and ‘buffered’ variants described earlier. A variant at the origin of replication of the mitochondrial heavy strand, C150T, has been observed at higher frequencies in centenarians, both through inheritance and through somatic increase in frequency, with some individuals achieving homoplasmy for this variant in their lymphocytes and monocytes, but not in granulocytes; a selective advantage of achieving high frequency of this variant in at least some cell types has been suggested (Zhang et al. 2003). Interactions between nuclear genome variants and both inherited and somatic mitochondrial variants have also been suggested to play a role in aging and longevity (Santoro et al. 2006; Tranah 2011).

### Genome sequencing

Sebastiani et al. (2011) recently reported the whole genome sequencing of one male and one female supercentenarian of European ancestry from the NECS. The genomes of these exceptionally long-lived individuals were similar, in terms of the rate of nonsynonymous SNPs and number of indels, to other genomes sequenced to date. They have a similar number of known disease-associated variants to other genomes showing that their exceptional lifespan does not seem to be due to lack of known disease-associated variants. It is possible, though, that they failed to inherit a combination of variants that would have acted together to cause disease. Both supercentenarians lacked *APOE4* alleles. They do not carry most of the longevity variants reported previously in the literature, implying that these known variants are not necessary for longevity. It is possible that they carry as yet undiscovered protective variants. One per cent of the variants observed were novel. Interestingly, an excess of coding region variants was seen in genes closest to GWAS-identified longevity variants, an observation that supports the idea that rare variants of these genes may contribute to the longevity phenotype.

### Telomeres in healthy aging and longevity

Telomeres are indisputably important to aging. Telomeres shorten with age and are considered to be a biomarker of age. The role of telomere biology in healthy aging and disease was recently reviewed (Zhu et al. 2011). Leukocyte telomere length (LTL) has been correlated with measures of health and ability in elderly individuals. In a community-based cohort of 70- to 79-year-olds, LTL was associated with more years of healthy life; LTL was suggested to be a biomarker of healthy aging (Njajou et al. 2009). Louisiana Healthy Aging Study results concurred with this observation; LTL was correlated with measures of healthy aging in an age-dependent way (Kim et al. 2012). LTL was also found to correlate positively with physical ability (but not cognitive function) in Danish twins aged at least 77 years (Bendix et al. 2011) and inversely with disability in American seniors (Risques et al. 2010). Ashkenazi centenarians and their offspring also showed longer telomeres, for their age, than controls; longer telomeres correlated with less disease (Atzmon et al. 2010). In contrast, in a study of Canadian ‘Super-Seniors’ (individuals aged at least 85 and never diagnosed with cancer, cardiovascular disease, Alzheimer disease, major pulmonary disease or diabetes) the healthy oldest-old did not have exceptional telomere length for their age, but showed less variability in telomere length than mid-life controls, implying that they may be selected for optimal rather than extreme telomere length (Halaschek-Wiener et al. 2008).

Variation in genes involved in telomere maintenance has also been associated with longevity. One SNP at *SIRT1* (Kim et al. 2012) and one in *TERC* (Soerensen et al. 2012) are associated with both LTL and longevity. Detailed analysis of* TERT* and* TERC* in Ashkenazi centenarians showed an excess of genetic variation in both genes in the centenarians and identified a* TERT* haplotype associated with extreme longevity (Atzmon et al. 2010). Gene set analysis of GWAS data also supported the relevance of telomere maintenance (Deelen et al. 2013). Overall, the relationship between telomeres, aging, healthy aging, and longevity is multi-layered. Telomere maintenance is an important process in aging, and also a biomarker of it. LTL is a biomarker of aging and of healthy aging. Variation in telomere maintenance genes appears to affect both telomere length, and life span and health span in humans.

### Somatic genetics of aging

Two recent large-scale analyses of data from GWAS studies have established that mosaicism for large genomic alterations increases with age (Laurie et al. 2012; Jacobs et al. 2012). In one study, data for 50,222 subjects found that <0.5 % of people aged <50, and 2–3 % of elderly (2.7 % in subjects >80 years), have detectable mosaicism in peripheral blood. Age was a significant predictor of mosaic status, but sex, ancestry, and smoking status were not. The second study used data from 31,717 cancer cases and 26,136 controls from 13 GWAS studies and found detectable clonal mosaicism in 0.87 % of individuals. In the cancer-free controls, they found mosaicism in 0.23 % of those <50 years old and in 1.91 % of those aged 75–79, a significant difference (*p* = 4.8 × 10^−8^). Somatic mosaicism (heteroplasmy) of the mitochondrial genome also increases over the lifespan (Sondheimer et al. 2011). Of course, telomere shortening is another somatic genomic change that occurs over the human lifespan. Such somatic changes are both a genetic aspect of aging and an aging-related phenotype.

### Epigenetics and longevity/aging

Epigenetics, at the interface between the genome and the environment, is emerging as an important factor in longevity, and has been the subject of recent excellent reviews (Gravina and Vijg 2010; Ben-Avraham et al. 2012). Methylation patterns change with age, and discordance in methylation between MZ twins also increases with age (Talens et al. 2012), an observation consistent with the effect of environment and lifestyle on the epigenome. Studies of DNA methylation support the idea that aging is associated with a relaxation of epigenetic control and that this epigenetic drift may affect the development of aging-related diseases (Gravina and Vijg 2010). An epigenome-wide association scan (EWAS) identified age-related differentially methylated regions as well as differentially methylated regions associated with age-related phenotypes (Bell et al. 2012). Whole genome bisulfite sequencing of DNA from CD4^+^ T cells of a centenarian and a newborn identified differentially methylated regions that were usually hypomethylated and less correlated with methylation of adjacent CpG dinucleotides in the centenarian (Heyn et al. 2012). These results support the idea that small cumulative DNA methylation changes accumulate over a lifetime. Age-related temporal changes in DNA methylation also show significant familial clustering, indicating that methylation maintenance is a familial trait (Bjornsson et al. 2008). A study of DNA methylation in centenarians and their offspring compared with the offspring of non-long-lived individuals and young individuals showed that the offspring of the centenarians delay age-related methylation changes (Gentilini 2012). A landmark paper by Hannum et al. (2013) offers an explanation for this familiality. They used methylome analysis to compare human aging rates in individuals of age 19–101 and identify methylation QTLs (meQTLs) (including one at methyl-CpG binding domain protein 4) that affect it. Indeed, trans-generational epigenetic inheritance of extended lifespan has been demonstrated in *C. elegans* (Greer et al. 2011).

It is likely that the effects of epigenetic changes manifest in part by effects on gene expression. Longevity-selected lines of Drosophila show gene expression profiles that are similar to younger control flies (Sarup et al. 2011). This type of observation is more difficult to make in humans, however. Several human studies have compared gene expression between LLI and younger individuals. Blood miRNA expression differences between LLI and younger controls identified genes known to be differentially expressed in age-related diseases (ElSharawy et al. 2012). This study design, however, does not allow discrimination between genes that are differentially expressed because they are involved in longevity, related to chronological age, or affected by environmental differences between the old and young groups. A cross-sectional analysis of individuals aged 50–90, and centenarians, was used to identify a miRNA, miR-363*, whose expression declined with age but was preserved at youthful levels in the centenarians (Gombar et al. 2012). The Leiden Longevity Study, however, used LLI and their offspring to show that *RPTOR* in the mTOR pathway is differentially expressed between the offspring of the LLI and their spouses (Passtoors et al. 2013). The study design issues that are important to avoid confounding by lifestyle factors in studies of inherited factors will be even more important in gene expression studies. It seems likely that as yet unidentified genetic factors and lifestyle practices that help us maintain a favorable epigenetic profile and optimal gene expression will be important in longevity and healthy aging.

## Conclusions

To date, studies of longevity and healthy aging have shown few consistent and many inconsistent results. This is probably due in part to the nature of the variants we seek to find, which may be rare or even private, may act in concert or as a ‘signature’, and which may buffer against the presence of other variants. It is also due to inter-ethnic differences, population stratification, differences in phenotype definition and study design, effects of and confounding by known and unrecognized non-genetic factors in part due to cohort effects, and exacerbation by insufficient sample size. Nevertheless, the results of longevity studies to date establish their importance for our understanding of health and disease.

Only one gene has emerged as consistently found in studies of LLI, *APOE*, though another, *FOXO3A*, was replicated in multiple candidate gene studies. Additional candidate genes are associated in more than one but not all studies. Many of these correspond to signals that hover below GWS in GWAS studies, and when analyzed together for commonalities of pathways or other processes by means such as gene set analysis, as a group are significantly associated with these phenotypes. Epigenetics is quickly emerging as a critical aspect of aging and longevity. Centenarians delay age-related methylation changes, and they can pass this methylation preservation ability on to their offspring, probably via genetic variants that affect methylation QTLs.

Importantly and perhaps surprisingly, centenarians appear to have the same numbers of GWAS-identified common disease variants as ordinary people, and yet they have lived long and, to a great extent, free of disease. Specific examples support the idea that centenarians may have advantageous alleles of ‘buffering’ genes that allow them to remain healthy despite the presence of ‘buffered’ deleterious alleles in other genes. It will be important to determine how generalizeable this is—does it apply only to a few genes, or is it a general mechanism for suppression of disadvantageous alleles of many genes? As others have pointed out (Bergman et al. 2007), a longevity gene that buffered frailty alleles in several other genes would be a desirable drug development tool. Understanding this will affect not only personalized medicine but also our overall interpretation of genomes, and could even give us the information needed for rational design of agents to re-create this desirable scenario in those not lucky enough to inherit it.
